# Supplementary material for: High-Throughput Drug Library Screening in Primary KMT2A-Rearranged Infant ALL Cells Favors the Identification of Drug Candidates That Activate P53 Signaling
Source: Biomedicines. 2022 Mar 10;10(3):638. doi: 10.3390/biomedicines10030638 (PMC8945716; doi:10.3390/biomedicines10030638)
Supplement: Supplementary file 1 [file biomedicines-10-00638-s001.zip › biomedicines-1595804-supplementary.pdf]

Figure S1

A

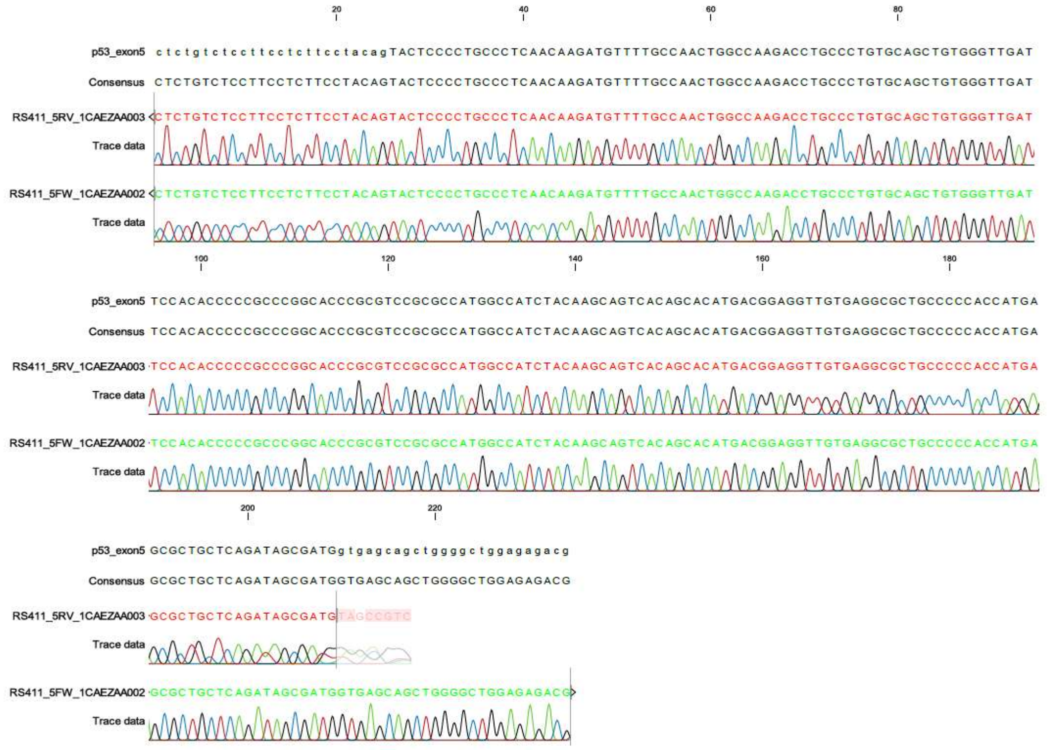

B

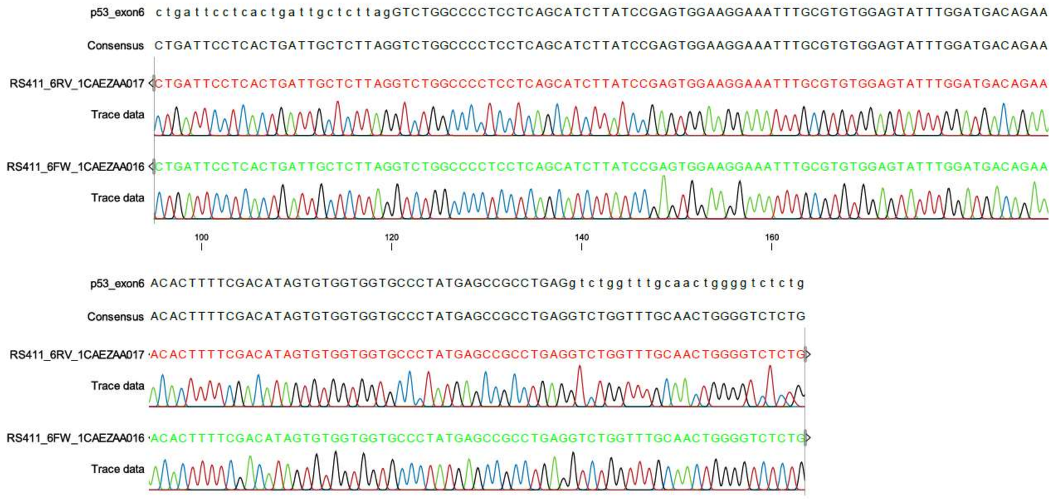

C

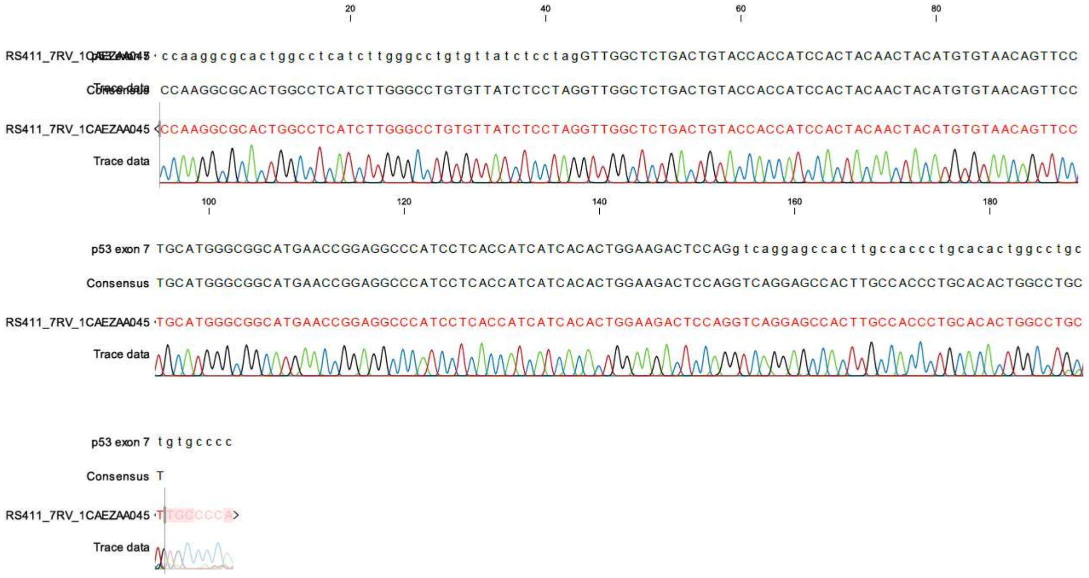

20
40
60
80
ccaaggcgcactggcctcatcttgggcctgtgttatctcctagGTTGGCTCTGACTGTACCACCATCCACTACAACTACATGTGTAACAGTTCC
CCAAGGCGCACTGGCCTCATCTTGGGCCTGTGTTATCTCCTAGGTTGGCTCTGACTGTACCACCATCCACTACAACTACATGTGTAACAGTTCC
RS411_7RV_1CAEZAA045
CCAAGGCGCACTGGCCTCATCTTGGGCCTGTGTTATCTCCTAGGTTGGCTCTGACTGTACCACCATCCACTACAACTACATGTGTAACAGTTCC
Trace data
100
120
140
160
180
p53 exon 7
TGCATGGGCGGCATGAACCGGAGGCCCATCCTCACCATCATCACACTGGAAGACTCCAGgtcaggagccacttgccaccctgcacactggcctgc
Consensus
TGCATGGGCGGCATGAACCGGAGGCCCATCCTCACCATCATCACACTGGAAGACTCCAGGTCAGGAGCCACTTGCCACCCTGCACACTGGCCTGC
RS411_7RV_1CAEZAA045
TGCATGGGCGGCATGAACCGGAGGCCCATCCTCACCATCATCACACTGGAAGACTCCAGGTCAGGAGCCACTTGCCACCCTGCACACTGGCCTGC
Trace data
p53 exon 7
tgtgcccc
Consensus
T
RS411_7RV_1CAEZAA045
Trace data

D

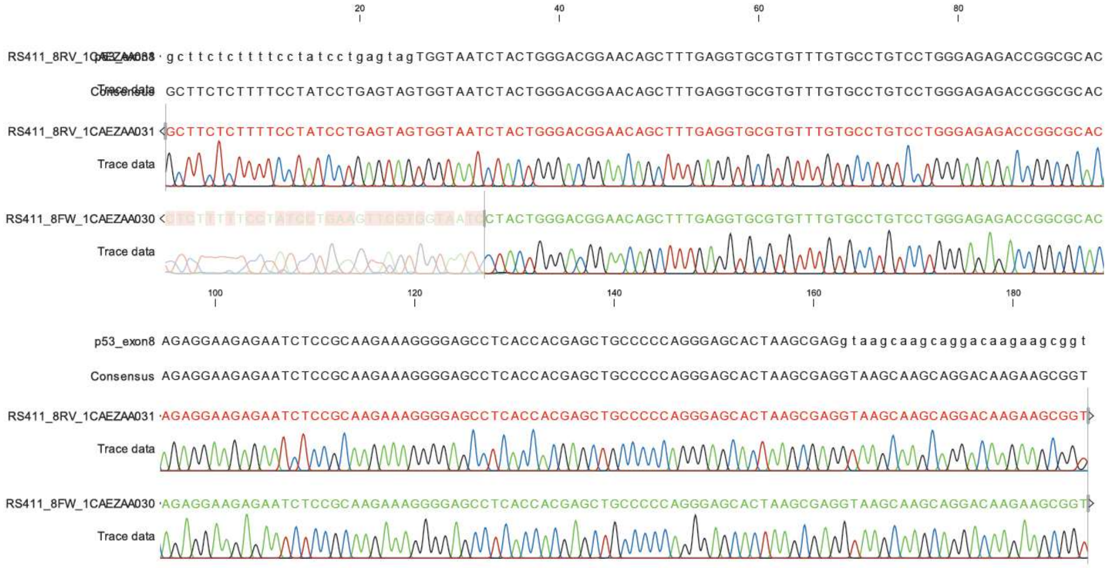

20
40
60
80
gcttctcttttcctatcctgagtagTGGTAATCTACTGGGACGGAACAGCTTTGAGGTGCGTGTTTGTGCCTGTCCTGGGAGAGACCGGCGCAC
GCTTCTCTTTTCCTATCCTGAGTAGTGGTAATCTACTGGGACGGAACAGCTTTGAGGTGCGTGTTTGTGCCTGTCCTGGGAGAGACCGGCGCAC
RS411_8RV_1CAEZAA031
GCTTCTCTTTTCCTATCCTGAGTAGTGGTAATCTACTGGGACGGAACAGCTTTGAGGTGCGTGTTTGTGCCTGTCCTGGGAGAGACCGGCGCAC
Trace data
RS411_8FW_1CAEZAA030
CTACTGGGACGGAACAGCTTTGAGGTGCGTGTTTGTGCCTGTCCTGGGAGAGACCGGCGCAC
Trace data
100
120
140
160
180
p53_exon8
AGAGGAAGAGAATCTCCGCAAGAAAGGGGAGCCTCACCACGAGCTGCCCCCAGGGAGCACTAAGCGAGgtaagcaagcaggacaagaagcggt
Consensus
AGAGGAAGAGAATCTCCGCAAGAAAGGGGAGCCTCACCACGAGCTGCCCCCAGGGAGCACTAAGCGAGGTAAGCAAGCAGGACAAGAAGCGGT
RS411_8RV_1CAEZAA031
AGAGGAAGAGAATCTCCGCAAGAAAGGGGAGCCTCACCACGAGCTGCCCCCAGGGAGCACTAAGCGAGGTAAGCAAGCAGGACAAGAAGCGGT
Trace data
RS411_8FW_1CAEZAA030
AGAGGAAGAGAATCTCCGCAAGAAAGGGGAGCCTCACCACGAGCTGCCCCCAGGGAGCACTAAGCGAGGTAAGCAAGCAGGACAAGAAGCGGT
Trace data

E

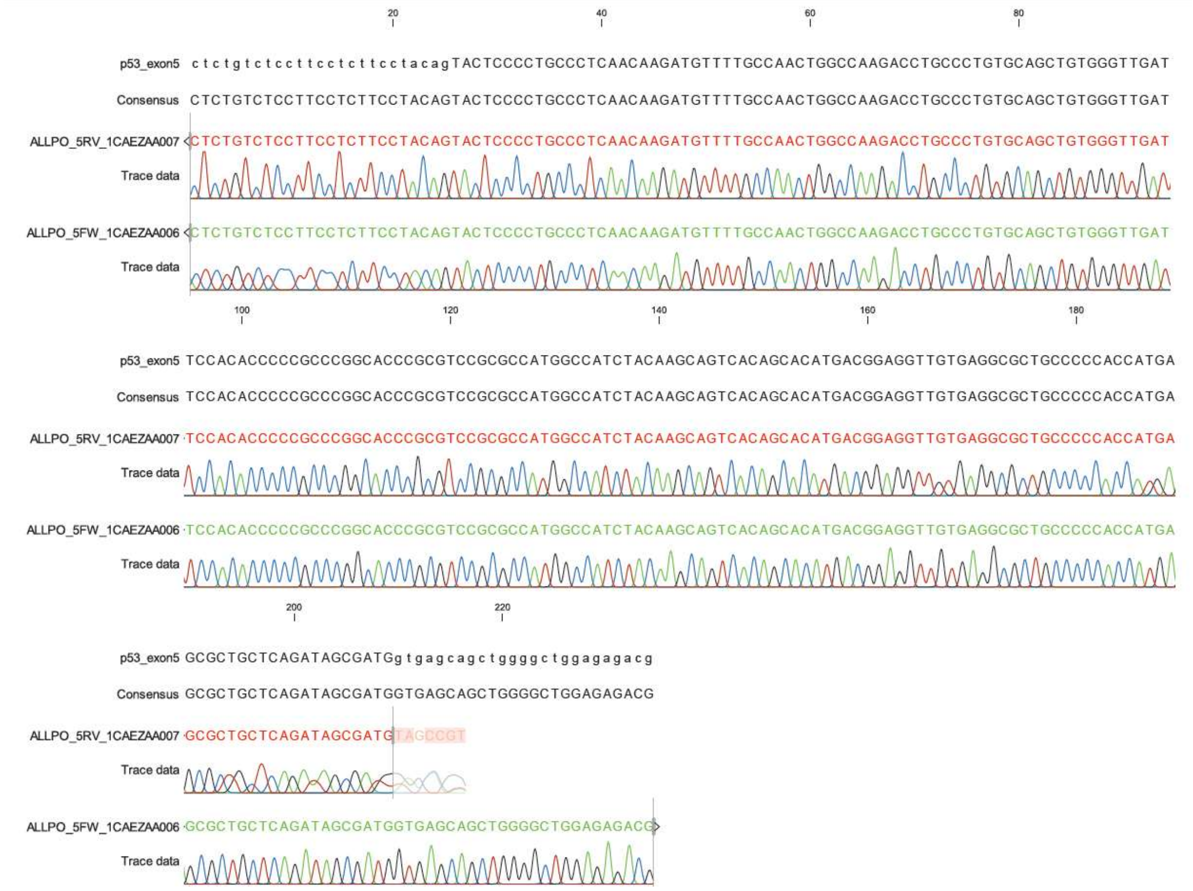
20
40
60
80
p53_exon5 ctctgtctccttcctcttcctacagTACTCCCCTGCCCTCAACAAGATGTTTTGCCAACTGGCCAAGACCTGCCCTGTGCAGCTGTGGGTTGAT
Consensus CTCTGTCTCCTTCCTCTTCCTACAGTACTCCCCTGCCCTCAACAAGATGTTTTGCCAACTGGCCAAGACCTGCCCTGTGCAGCTGTGGGTTGAT
ALLPO_5RV_1CAEZAA007 CTCTGTCTCCTTCCTCTTCCTACAGTACTCCCCTGCCCTCAACAAGATGTTTTGCCAACTGGCCAAGACCTGCCCTGTGCAGCTGTGGGTTGAT
Trace data
ALLPO_5FW_1CAEZAA006 CTCTGTCTCCTTCCTCTTCCTACAGTACTCCCCTGCCCTCAACAAGATGTTTTGCCAACTGGCCAAGACCTGCCCTGTGCAGCTGTGGGTTGAT
Trace data
100
120
140
160
180
p53_exon5 TCCACACCCCCGCCCGGCACCCGCGTCCGCGCCATGGCCATCTACAAGCAGTCACAGCACATGACGGAGGTTGTGAGGCGCTGCCCCCACCATGA
Consensus TCCACACCCCCGCCCGGCACCCGCGTCCGCGCCATGGCCATCTACAAGCAGTCACAGCACATGACGGAGGTTGTGAGGCGCTGCCCCCACCATGA
ALLPO_5RV_1CAEZAA007 TCCACACCCCCGCCCGGCACCCGCGTCCGCGCCATGGCCATCTACAAGCAGTCACAGCACATGACGGAGGTTGTGAGGCGCTGCCCCCACCATGA
Trace data
ALLPO_5FW_1CAEZAA006 TCCACACCCCCGCCCGGCACCCGCGTCCGCGCCATGGCCATCTACAAGCAGTCACAGCACATGACGGAGGTTGTGAGGCGCTGCCCCCACCATGA
Trace data
200
220
p53_exon5 GCGCTGCTCAGATAGCGATGgtgagcagctggggctggagagacg
Consensus GCGCTGCTCAGATAGCGATGGTGAGCAGCTGGGGCTGGAGAGACG
ALLPO_5RV_1CAEZAA007 GCGCTGCTCAGATAGCGATGTAGCCGT
Trace data
ALLPO_5FW_1CAEZAA006 GCGCTGCTCAGATAGCGATGGTGAGCAGCTGGGGCTGGAGAGACG
Trace data

F

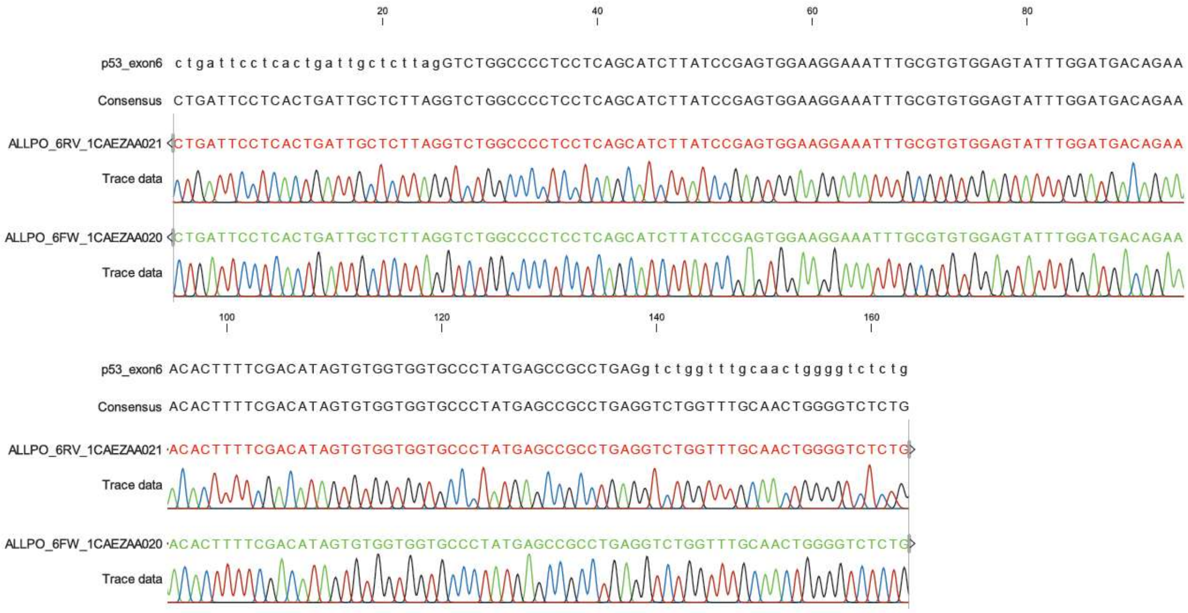
20
40
60
80
p53_exon6 ctgattcctcactgattgctcttagGTCTGGCCCCTCCTCAGCATCTTATCCGAGTGGAAGGAAATTTGCGTGTGGAGTATTTGGATGACAGAA
Consensus CTGATTCCTCACTGATTGCTCTTAGGTCTGGCCCCTCCTCAGCATCTTATCCGAGTGGAAGGAAATTTGCGTGTGGAGTATTTGGATGACAGAA
ALLPO_6RV_1CAEZAA021 CTGATTCCTCACTGATTGCTCTTAGGTCTGGCCCCTCCTCAGCATCTTATCCGAGTGGAAGGAAATTTGCGTGTGGAGTATTTGGATGACAGAA
Trace data
ALLPO_6FW_1CAEZAA020 CTGATTCCTCACTGATTGCTCTTAGGTCTGGCCCCTCCTCAGCATCTTATCCGAGTGGAAGGAAATTTGCGTGTGGAGTATTTGGATGACAGAA
Trace data
100
120
140
160
p53_exon6 ACACTTTTCGACATAGTGTGGTGGTGCCCTATGAGCCGCCTGAGgtctggtttgcaactggggtctctg
Consensus ACACTTTTCGACATAGTGTGGTGGTGCCCTATGAGCCGCCTGAGGTCTGGTTTGCAACTGGGGTCTCTG
ALLPO_6RV_1CAEZAA021 ACACTTTTCGACATAGTGTGGTGGTGCCCTATGAGCCGCCTGAGGTCTGGTTTGCAACTGGGGTCTCTG
Trace data
ALLPO_6FW_1CAEZAA020 ACACTTTTCGACATAGTGTGGTGGTGCCCTATGAGCCGCCTGAGGTCTGGTTTGCAACTGGGGTCTCTG
Trace data

G

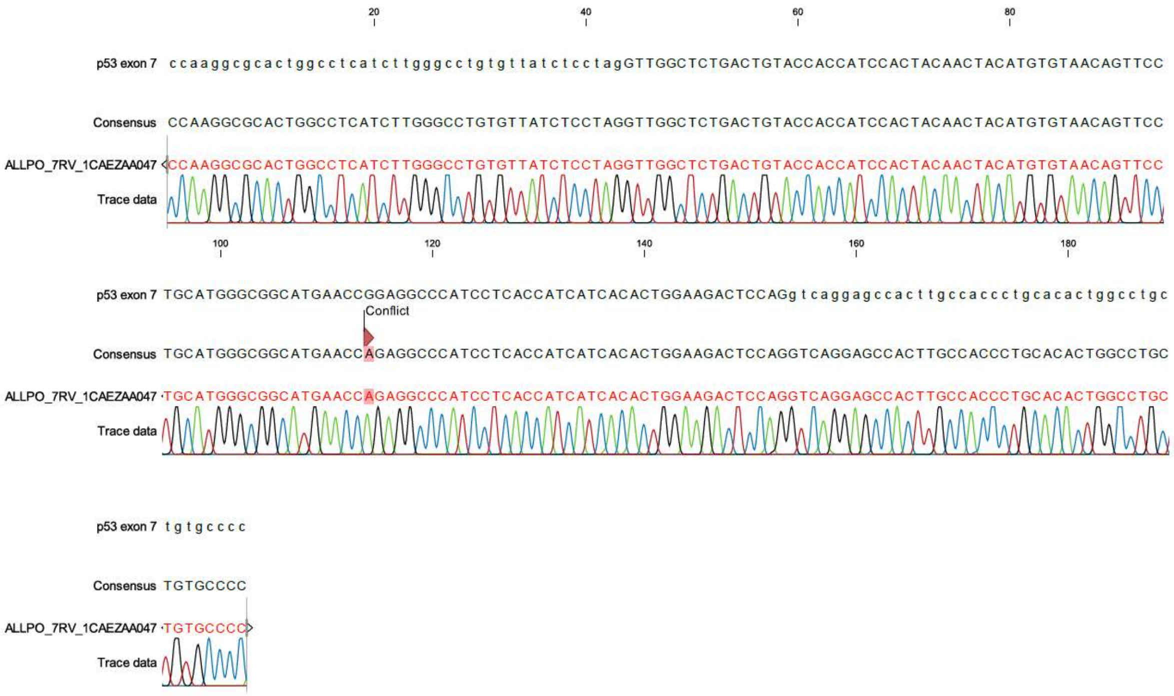
20
40
60
80
p53 exon 7 ccaaggcgcactggcctcatcttgggcctgtgttatctcctagGTTGGCTCTGACTGTACCACCATCCACTACAACTACATGTGTAACAGTTCC
Consensus CCAAGGCGCACTGGCCTCATCTTGGGCCTGTGTTATCTCCTAGGTTGGCTCTGACTGTACCACCATCCACTACAACTACATGTGTAACAGTTCC
ALLPO_7RV_1CAEZAA047 CCAAGGCGCACTGGCCTCATCTTGGGCCTGTGTTATCTCCTAGGTTGGCTCTGACTGTACCACCATCCACTACAACTACATGTGTAACAGTTCC
Trace data
100
120
140
160
180
p53 exon 7 TGCATGGGCGGCATGAACCGGAGGCCCATCCTCACCATCATCACACTGGAAGACTCCAGgtcaggagccacttgccaccctgcacactggcctgc
Conflict
Consensus TGCATGGGCGGCATGAACCAGAGGCCCATCCTCACCATCATCACACTGGAAGACTCCAGGTCAGGAGCCACTTGCCACCCTGCACACTGGCCTGC
ALLPO_7RV_1CAEZAA047 TGCATGGGCGGCATGAACCAGAGGCCCATCCTCACCATCATCACACTGGAAGACTCCAGGTCAGGAGCCACTTGCCACCCTGCACACTGGCCTGC
Trace data
p53 exon 7 tgtgcccc
Consensus TGTGCCCC
ALLPO_7RV_1CAEZAA047 TGTGCCCC
Trace data

H

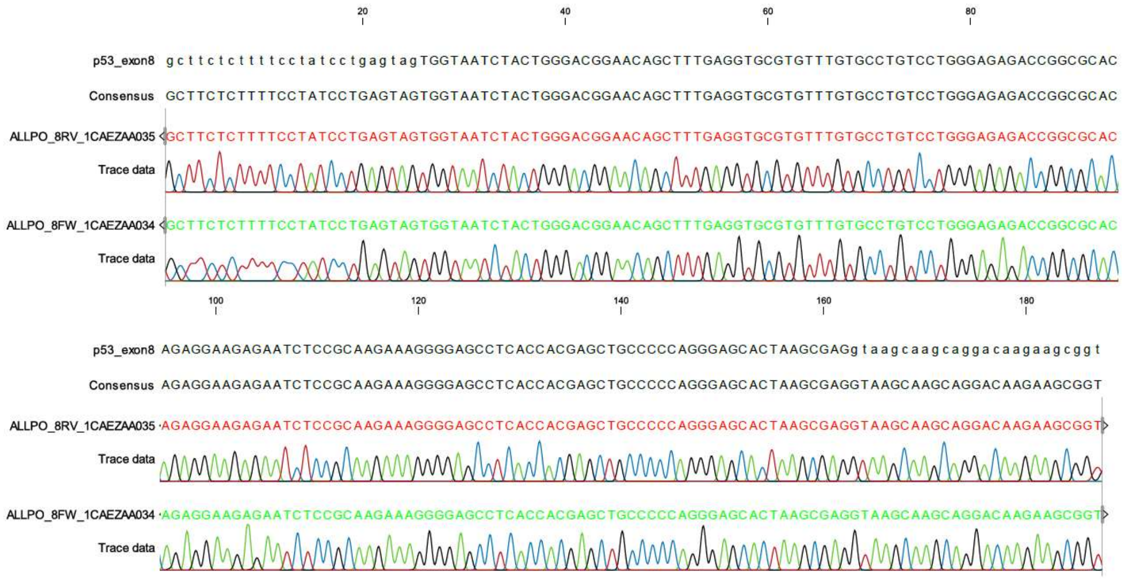
20
40
60
80
p53_exon8 gcttctcttttcctatcctgagtagTGGTAATCTACTGGGACGGAACAGCTTTGAGGTGCGTGTTTGTGCCTGTCCTGGGAGAGACCGGCGCAC
Consensus GCTTCTCTTTTCCTATCCTGAGTAGTGGTAATCTACTGGGACGGAACAGCTTTGAGGTGCGTGTTTGTGCCTGTCCTGGGAGAGACCGGCGCAC
ALLPO_8RV_1CAEZAA035 GCTTCTCTTTTCCTATCCTGAGTAGTGGTAATCTACTGGGACGGAACAGCTTTGAGGTGCGTGTTTGTGCCTGTCCTGGGAGAGACCGGCGCAC
Trace data
ALLPO_8FW_1CAEZAA034 GCTTCTCTTTTCCTATCCTGAGTAGTGGTAATCTACTGGGACGGAACAGCTTTGAGGTGCGTGTTTGTGCCTGTCCTGGGAGAGACCGGCGCAC
Trace data
100
120
140
160
180
p53_exon8 AGAGGAAGAGAATCTCCGCAAGAAAGGGGAGCCTCACCACGAGCTGCCCCCAGGGAGCACTAAGCGAGgtaagcaagcaggacaagaagcggt
Consensus AGAGGAAGAGAATCTCCGCAAGAAAGGGGAGCCTCACCACGAGCTGCCCCCAGGGAGCACTAAGCGAGGTAAGCAAGCAGGACAAGAAGCGGT
ALLPO_8RV_1CAEZAA035 AGAGGAAGAGAATCTCCGCAAGAAAGGGGAGCCTCACCACGAGCTGCCCCCAGGGAGCACTAAGCGAGGTAAGCAAGCAGGACAAGAAGCGGT
Trace data
ALLPO_8FW_1CAEZAA034 AGAGGAAGAGAATCTCCGCAAGAAAGGGGAGCCTCACCACGAGCTGCCCCCAGGGAGCACTAAGCGAGGTAAGCAAGCAGGACAAGAAGCGGT
Trace data

Figure S2

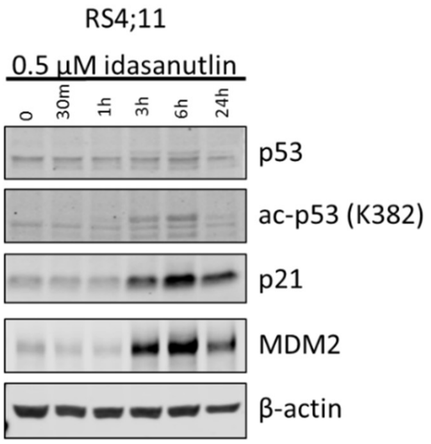

Figure S3

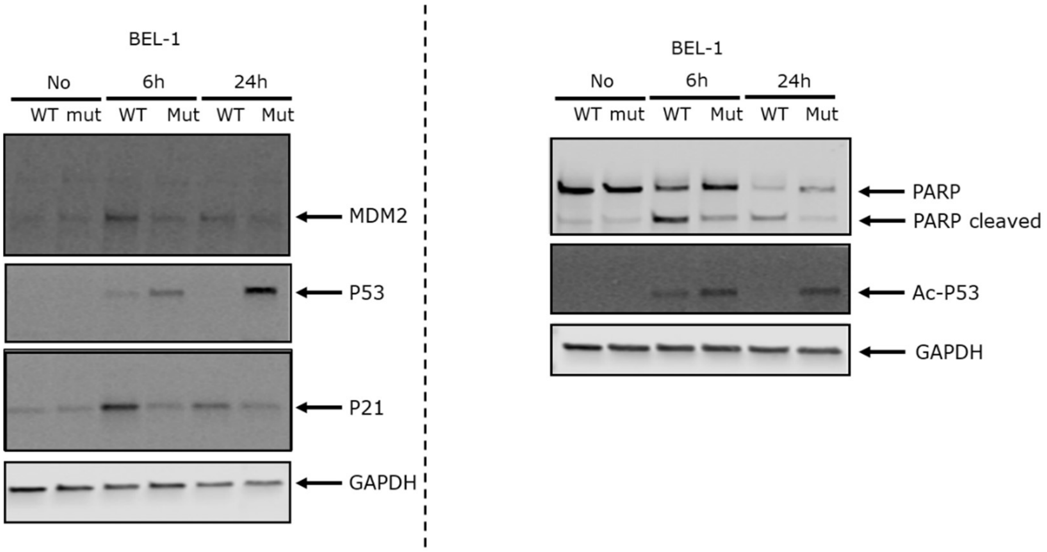

Figure S4

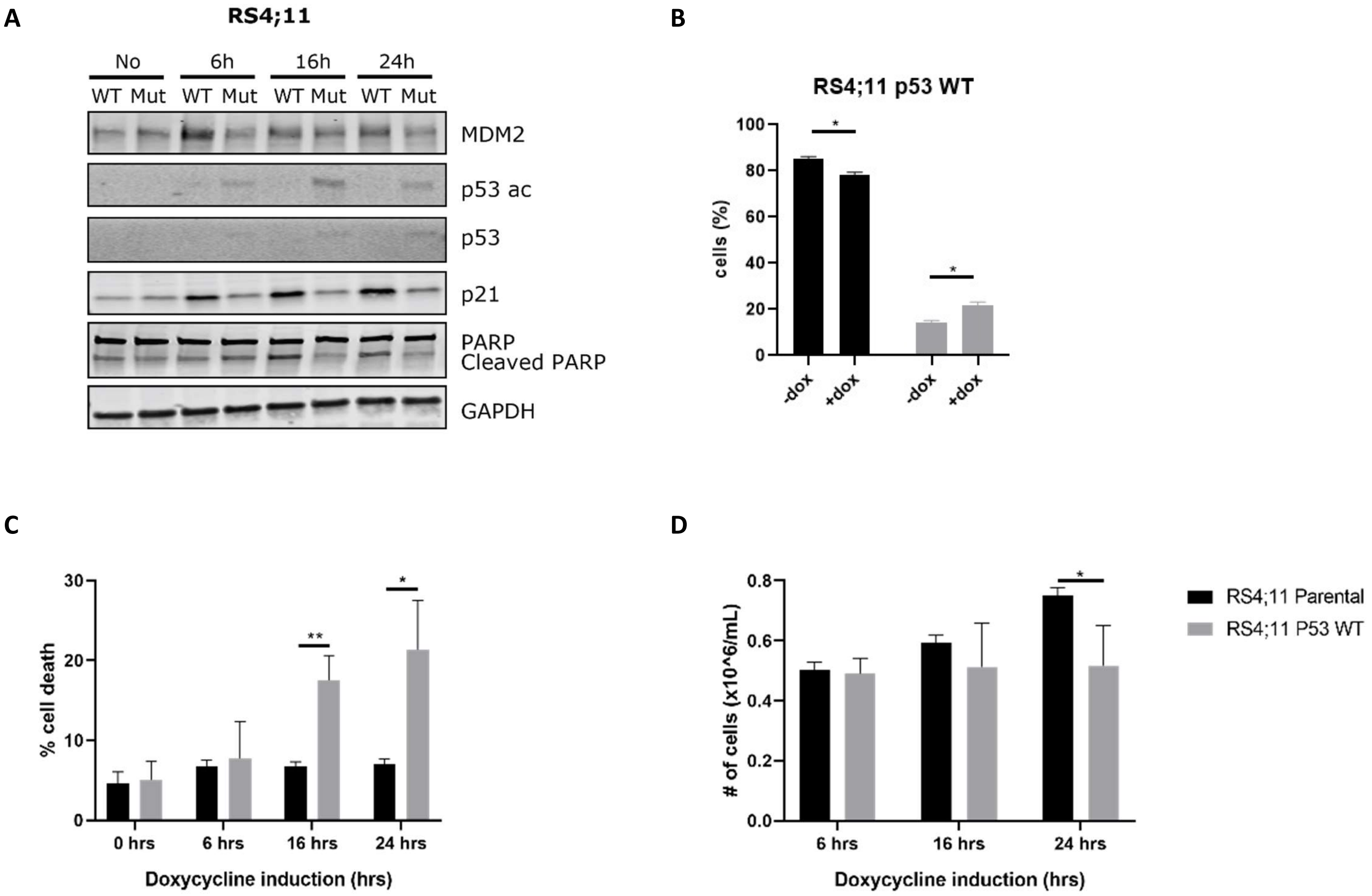
A
RS4;11
No
6h
16h
24h
WT Mut WT Mut WT Mut WT Mut
MDM2
p53 ac
p53
p21
PARP
Cleaved PARP
GAPDH
B
RS4;11 p53 WT
cells (%)
0
20
40
60
80
100
-dox
+dox
-dox
+dox
C
% cell death
0
10
20
30
0 hrs
6 hrs
16 hrs
24 hrs
Doxycycline induction (hrs)
D
# of cells (x10^6/mL)
0.0
0.2
0.4
0.6
0.8
6 hrs
16 hrs
24 hrs
Doxycycline induction (hrs)
RS4;11 Parental
RS4;11 P53 WT

Figure S5

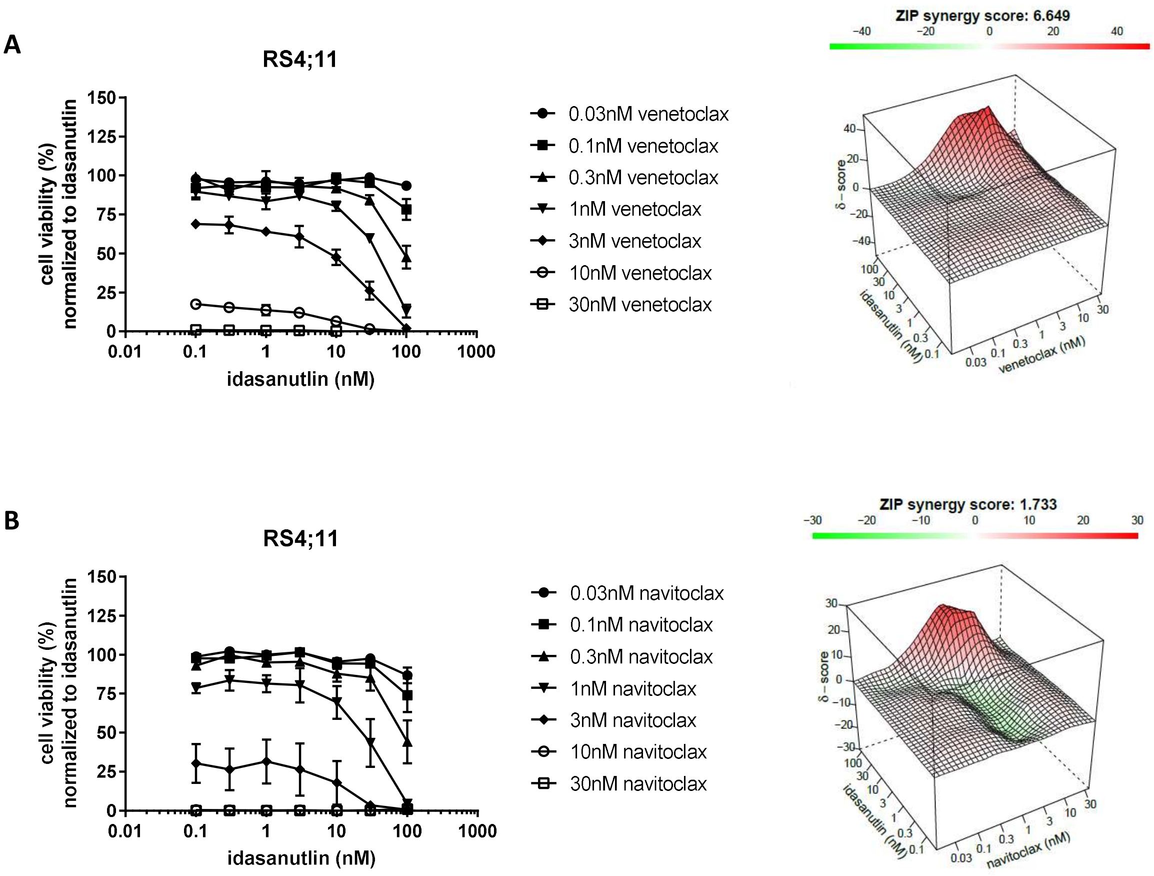
A
RS4;11
cell viability (%) normalized to idasanutlin
idasanutlin (nM)
0.03nM venetoclax
0.1nM venetoclax
0.3nM venetoclax
1nM venetoclax
3nM venetoclax
10nM venetoclax
30nM venetoclax
ZIP synergy score: 6.649
δ – score
idasanutlin (nM)
venetoclax (nM)
B
RS4;11
cell viability (%) normalized to idasanutlin
idasanutlin (nM)
0.03nM navitoclax
0.1nM navitoclax
0.3nM navitoclax
1nM navitoclax
3nM navitoclax
10nM navitoclax
30nM navitoclax
ZIP synergy score: 1.733
δ – score
idasanutlin (nM)
navitoclax (nM)

Supplementary Figure Legends

Figure S1. Hot spot mutation analysis reveals that cell line RS4;11 carries a wild-type *p53* and ALL-PO carries a missense mutation exon 7 of the p53 gene. Hot spot mutation analysis of p53 in RS4;11 in (A) exon 5, (B) exon 6, (C) exon 7 and (D) exon 8. Mutation analysis of *p53* in ALL-PO in (E) exon 5, (F) exon 6, (G) exon 7 and (H) exon 8. Data shown is based on Sanger sequencing. Genomic DNA was isolated from each cell line using the DNeasy Blood & Tissue kit (Qiagen) according to the manufacturer's instructions. p53 mutational hotspots were amplified by PCR using the following primers: Exon 5 FW-CTCTGTCTCCTTCCTCTTCCTACAG; exon 5 RV-CGTCTCTCCAGCCCCAGCTGCTCAC; exon 6 FW-CTGATTCCTCACTGATTGCTCTTAG; exon 6 RV-CAGAGACCCCAGTTGCAAACCAGAC; exon 7 FW-CCAAGGCGCACTGGCCTCATCTTGGGCCTGTGTTATCTCCTAG; exon 7 RV-GGGGCACAGCAGGCCAGTGTGCAGGGTGGCAAGTGGCTCCTGAC; exon 8 FW-GCTTCTCTTTTCCTATCCTGAGTAG; exon 8 RV-ACCGCTTCTTGTCCTGCTTGCTTA, with PCR program: 2 min on 50℃; 10 min on 95℃; 15 sec on 95℃ followed by 1 min on 60℃ for 40 cycles. PCR Products were verified by gel electrophoresis and isolated using the QIAquick Gel Extraction kit (Qiagen) prior to sending the DNA out for Sanger sequencing, which was performed at the Utrecht sequencing facility.

Figure S2. Time course analysis on the protein expression of the p53 pathway in presence of Idasanutlin in *KMT2A-AFF1* ALL cells. Western blot analysis on the expression of p53, p53 acetylation at lysine 382, target gene p21 and MDM2 upon exposure to 0.5µM Idasanutlin in RS4;11 over a time course ranging from 30 minutes to 24 hours. β-actin (Abcam) was used as loading control.

Figure S3. Effects of forced p53 activity on the expression of the p53 pathway in BEL-1 carrying either wild-type or mutated *p53*. Western blot analysis showing the protein expression of MDM2, p53 acetylation at lysine 382, p53, p21 and cleaved PARP measured at 6 and 24 hours post doxycycline induction in the transduced BEL-1 cell line carrying doxycycline-inducible expression vectors either encoding wild-type or mutated (R248Q) *p53*. Housekeeping gene GAPDH was used as loading control. Note, this figure is a biological replicate of Figure 4A.

Figure S4. Forced p53 activation mildly induces apoptosis in RS4;11 cells carrying wild-type *p53*. (A) Western blot analysis showing the protein expression of MDM2, p53 acetylation at lysine 382, p53, p21 and cleaved PARP measured at 6, 16 and 24 hours post doxycycline induction in the transduced RS4;11 cell line carrying doxycycline-inducible expression vectors either encoding wild-type or mutated (R248Q) p53. Housekeeping gene GAPDH was used as loading control. (B) Percentage of viable and apoptotic cells in RS4;11 cells carrying no vector (parental) or wild-type *p53*, at 24 hours post-p53 induction by doxycycline determined by flow cytometric analysis of the Annexin V/7AAD staining. (C) Percentage of dead cells and (D) percentage of viable cells, measured by a manual cell count of Trypan Blue positive cells RS4;11 cells and Trypan Blue negative RS4;11 cells carrying either no vector (parental) or wild-type *p53*, measured at 6, 16 and 24 hours post-p53 induction by doxycycline. All data points are represented as the mean ± SEM of n=3.

Figure S5. Idasanutlin and BCL2 inhibitors act in a synergistic manner on *KMT2A-AFF1* ALL cells. (A) *in vitro* cytotoxicity of Idasanutlin in combination with Venetoclax in RS4;11 cells (left graph) and the corresponding 3D synergy matrix (right graph). (B) *in vitro* cytotoxicity of Idasanutlin in combination with Navitoclax in RS4;11 cells (left graph) and the corresponding 3D synergy matrix (right graph). Cell viability is based on a 4-day MTT assay. Drug synergy was calculated using the Zero Interaction Potency (ZIP) scoring model in SynergyFinder [54] and normalized to the effect of Idasanutlin as single agent. Concentrations where Idasanutlin as single agent showed >70% cell toxicity on average were excluded from the synergy analysis. Data points are represented as the mean ± SEM of n=2 independent replicates.
